# Upregulated expression of FGF13/FHF2 mediates resistance to platinum drugs in cervical cancer cells

**DOI:** 10.1038/srep02899

**Published:** 2013-10-11

**Authors:** Tomoko Okada, Kazuhiro Murata, Ryoma Hirose, Chie Matsuda, Tsunehiko Komatsu, Masahiko Ikekita, Miyako Nakawatari, Fumiaki Nakayama, Masaru Wakatsuki, Tatsuya Ohno, Shingo Kato, Takashi Imai, Toru Imamura

**Affiliations:** 1 Signaling Molecules Research Group, Biomedical Research Institute, National Institute of Advanced Industrial Science and Technology (AIST), Tsukuba, Ibaraki 305-8566, Japan; 2 Division of Hematology, 3rd Department of Internal Medicine, Teikyo University Chiba Medical Center, Ichihara, Chiba 299-0111, Japan; 3 Department of Applied Biological Science, Faculty of Science and Technology, Tokyo University of Science, Noda, Chiba 278-8510, Japan; 4 Advanced Radiation Biology Research Program, National Institute of Radiological Sciences, Chiba, Chiba 263-8555, Japan; 5 Research Center Hospital for Charged Particle Therapy, National Institute of Radiological Sciences, Chiba, Chiba 263-8555, Japan; 6 Gunma University Graduate School of Medicine, Maebashi, Gunma 371-8511, Japan; 7 Saitama Medical University International Medical Center, Hidaka, Saitama 350-1298, Japan

## Abstract

Cancer cells often develop drug resistance. In cisplatin-resistant HeLa cisR cells, fibroblast growth factor 13 (FGF13/FHF2) gene and protein expression was strongly upregulated, and intracellular platinum concentrations were kept low. When the FGF13 expression was suppressed, both the cells' resistance to platinum drugs and their ability to keep intracellular platinum low were abolished. Overexpression of FGF13 in parent cells led to greater resistance to cisplatin and reductions in the intracellular platinum concentration. These cisplatin-resistant cells also showed increased resistance to copper. In preoperative cervical cancer biopsy samples from poor prognoses patients after cisplatin chemoradiotherapy, FGF13-positive cells were detected more abundantly than in the biopsy samples from patients with good prognoses. These results suggest that FGF13 plays a pivotal role in mediating resistance to platinum drugs, possibly via a mechanism shared by platinum and copper. Our results point to FGF13 as a novel target and useful prognostic guide for cancer therapy.

FGF13/FHF2 is one of the fibroblast growth factor (FGF) homologous factors (FHFs) present in many vertebrate species. These proteins bear strong sequence and structural similarity to FGFs and have therefore also been designated FGF12 (FHF1), FGF13 (FHF2), FGF11 (FHF3) and FGF14 (FHF4)^1^,^2^,^3^. However, FHFs lack N-terminal secretion signal sequences, are not secreted via a classical pathway from expressing cells, and cannot interact with FGF receptor tyrosine kinases. The C-terminal tails of FHFs are composed of approximately 40 amino acids that contribute to the proteins' ability to bind to and modulate voltage-gated sodium channels (VGSCs) and the MAP kinase scaffold protein islet brain 2 (IB2)^4^. It was also recently reported that FGF13 is a microtubule-stabilizing protein that regulates neuronal polarization and migration^5^. Thus, FHFs are intracellular proteins with activity profiles that differ substantively from those of other FGF family members^6^.

Cisplatin is a platinum-containing, very active anticancer agent that shows relevant clinical activity against a wide variety of human solid tumors^7^,^8^,^9^. Like two other platinum-containing anticancer drugs, carboplatin and oxaliplatin, cisplatin is incorporated into cells, where it induces formation of a platinum-DNA complex in the nucleus, thereby activating several processes that mediate cytotoxicity. However, cancer cells often develop resistance to cisplatin, which hampers effective chemotherapy. Several mechanisms for cisplatin resistance have been proposed, including decreased drug uptake, increased drug efflux, increased detoxification via the glutathione or metallothionein system, decreased DNA platination and increased DNA repair^10^,^11^,^12^,^13^. In this report we show that FGF13/FHF2 plays a pivotal role in cellular platinum drug resistance and in reducing intracellular platinum concentrations in cancer cells. Thus, we believe our report to be the first to document a clear biological function of FGF13/FHF2 in drug resistance.

## Results

### HeLa cisR and S180 cells, sublines resistant to cisplatin and its derivatives, express upregulated levels of FGF13

**Figure 1:**
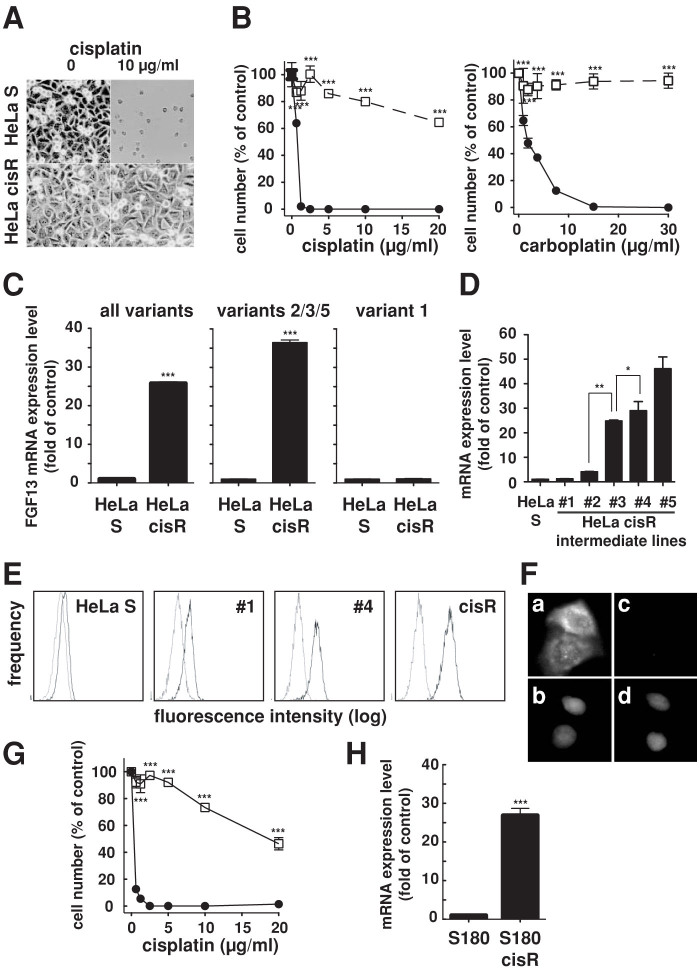
Expression of FGF13 mRNAs is upregulated in HeLa cisR and S180 cisR cells. (A). Phase contrast micrographs showing HeLa S and HeLa cisR cells cultured for 3 days in the absence or presence of cisplatin (10 μg/ml). (B). Effects of cisplatin and carboplatin on proliferation measured by MTT-assay. HeLa cisR, open squares; HeLa S, filled circles. ***, p < 0.001. (C). Levels of FGF13 mRNA expression detected by quantitative RT-PCR presented as fold increases over control. ***, p < 0.001. (D). FGF13 mRNA expression presented as fold changes from HeLa S cells. Five intermediate cell lines (#1 to #5, adapted to 0.42, 0.84, 2.0, 6.0 and 8.0 μg/ml cisplatin) were collected during the process of establishing the HeLa cisR. **, p < 0.01; *, p < 0.05. (E). FACS analysis of FGF13 expression. Solid line, treated with anti-FGF13 antibody; dotted line, without anti-FGF13 antibody. (F). Immunofluorescence analysis of FGF13 in HeLa S cells transiently transfected with FGF13-expression vector (a) or mock vector (c). Nuclei were counterstained with propidium iodide (b and d). (G). Effects of cisplatin on the proliferation. S180 cisR, open squares; S180 cells, filled circles. ***, p < 0.001. (H). FGF13 mRNA expression in S180 cisR. ***, p < 0.001.

**Figure 2:**
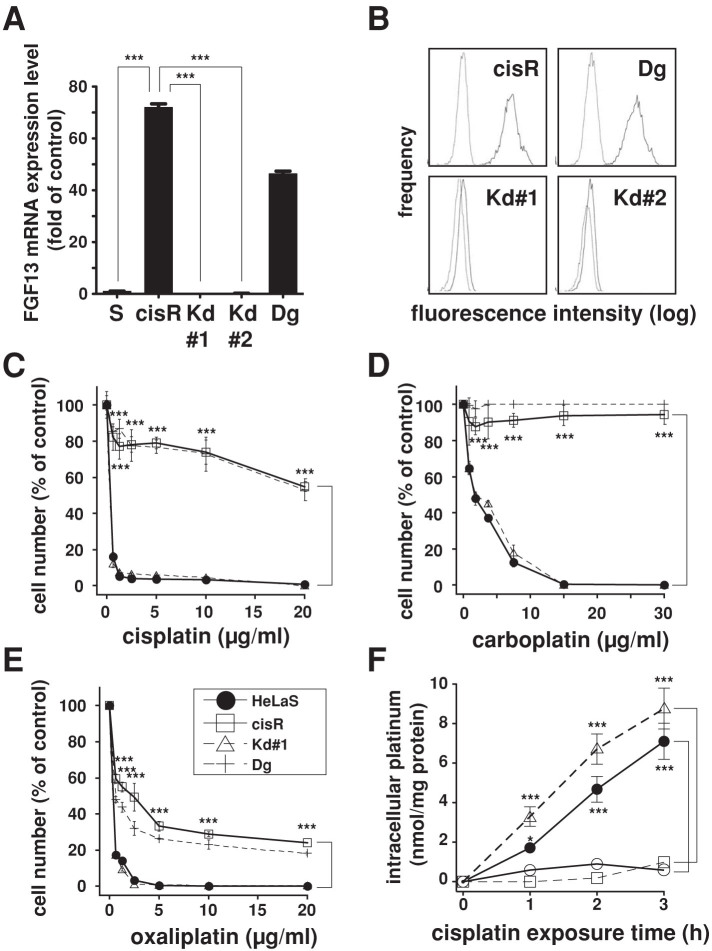
Knocking down FGF13 expression restores platinum drug susceptibility to HeLa cisR cells. (A). FGF13 mRNA expression. HeLa cisR cells were transfected with RNAi expression vector and cloned to yield FGF13Kd#1, Kd#2 and mock transfectant. ***, p < 0.001. (B). FACS analysis of FGF13 expression. Solid line, treated with anti-FGF13 antibody; dotted line, without anti-FGF13 antibody. (C), (D), (E). Suppressing FGF13 expression made HeLa cisR cells susceptible to cisplatin (C), carboplatin (D) and oxaliplatin (E). HeLa cisR, open squares solid line; HeLa S, filled circles solid line; FGF13Kd#1, open triangles dotted line; HeLa cisR with degenerate RNAi, crosses dotted line. ***, p < 0.001. (F). Intracellular platinum concentrations after cells were exposed to 30 μg/ml cisplatin and sonicated, measured by ICP Atomic Emission Spectrometry. HeLa cisR, open squares; HeLa S, filled circles; FGF13Kd#1, open triangles dotted line; mock HeLa cisR transfectants, open circles. ***, p < 0.001; *, p < 0.05.

We established a cisplatin-resistant HeLa cisR subline by gradually increasing the concentration of cisplatin in their growth medium. The numbers of HeLa cisR cells after culture for 3 days in medium containing 10 μg/ml cisplatin often reached 80% of the numbers seen in control cultures without cisplatin (Fig. 1A and B). By examining a wider range of cisplatin concentrations, we determined the 50% inhibitory concentration (IC_50_) of cisplatin for HeLa cisR cells to be 29.7 μg/ml, or about 60 times higher than for the parent HeLa S cells (0.49 μg/ml). HeLa cisR cells were also more resistant to the cisplatin derivatives carboplatin (Fig. 1B) and oxaliplatin (Fig. 2E) than were the parent cells.

To better understand the molecular mechanism underlying cisplatin resistance in HeLa cisR cells, we used DNA oligonucleotide microarrays to preliminarily screen for genes that showed large differences in their expression levels between resistant HeLa cisR and nonresistant HeLa S parent cells. FGF13 was identified as one such gene (Supplementary Table S1: The genes listed showed upregulated expression in HeLa cisR cells as compared to HeLa S cells). Using quantitative RT-PCR, we confirmed that expression of FGF13 mRNA in HeLa cisR cells was more than 25-fold higher than in HeLa S cells (Fig. 1C, a sequence common to all variants was measured; Table 2). FGF13 mRNA is reportedly expressed as several splice variants in the central nervous system^14^. We found that expression of FGF13 mRNA variants 2, 3 and 5 was strongly upregulated (approximately 36 times) in HeLa cisR cells (Fig. 1C). Variant 1 was expressed at a low level in the parent HeLa S cells and was not upregulated in HeLa cisR cells (Fig. 1C), while variants 4 and 6 were not expressed at detectable levels (not shown). In addition, HeLa S cells collected at intermediate times during the establishment of cisR cells exhibited intermediate levels of resistance to cisplatin and incrementally upregulated levels of FGF13 mRNA expression (Fig. 1D). The upregulated expression of FGF13 protein in cisplatin-resistant cell lines was confirmed by flow cytometry using an anti-FGF13 antibody (Fig. 1E). The specific reactivity of this antibody toward FGF13 protein was validated by comparing HeLa S cells transiently transfected with an FGF13 expression vector (Fig. 1Fa) to cells transfected with a mock vector (Fig. 1Fc). The specific reactivity of the antibody was then further confirmed through Western blot analysis of total proteins extracted from HEK cells transfected with an FGF13 expression vector (Supplementary Fig. S1).

We then examined S180 cisR cells, a previously established cisplatin-resistant mouse sarcoma cell line^15^, which also can survive in the presence of very high concentrations of cisplatin (Fig. 1G). Despite the differences in cancer type and species between HeLa cisR and S180 cisR cells, expression of mouse FGF13 mRNA was strongly upregulated in S180 cisR cells (Fig. 1H), just as human FGF13 mRNA was upregulated in HeLa cisR cells.

### Suppression of FGF13 expression in HeLa cisR cells abolishes the platinum drug resistance and increases the intracellular platinum concentration

To assess the potential involvement of upregulated FGF13 expression in the cisplatin resistance of HeLa cisR cells, we established two stable HeLa cisR FGF13-knockdown clones (Kd#1 and Kd#2) in which FGF13 mRNA expression (all variants) was selectively suppressed by RNA interference (Fig. 2A). The FGF13 knockdown was also confirmed at the protein level using flow cytometry (Fig. 2B).

Notably, knockdown clone Kd#1 was susceptible to the cytotoxicity of cisplatin (Fig. 2C), carboplatin (Fig. 2D) and oxaliplatin (Fig. 2E), and knockdown clone Kd#2 showed essentially the same susceptibility (Supplementary Figure S2). Thus, suppression of FGF13 expression in HeLa cisR cells abolished their platinum drug resistance. By contrast, control cells transfected with a degenerate RNAi sequence showed strong resistance to platinum drugs, as did HeLa cisR cells (Fig. 2C, D, E).

When FGF13 knockdown cells (Kd#1) were exposed to medium containing a high concentration (30 μg/ml) of cisplatin, intracellular platinum levels rapidly increased, just as in the parent HeLa S cells (Fig. 2F). In intact HeLa cisR cells, by contrast, intracellular platinum levels were kept very low (Fig. 2F), which is consistent with their survival in the presence of high cisplatin concentrations (10–20 μg/ml; Fig. 1B). Control cells transfected with a degenerate RNAi sequence showed strong FGF13 expression (Fig. 2A, B) and were capable of maintaining low intracellular platinum levels (Fig. 2F). We also found that the FGF13-dependent suppression of intracellular platinum levels was ongoing from the beginning (1 h post-exposure) of the incubation. Thus upregulated expression of FGF13 appears to be crucial for HeLa cell resistance to platinum anticancer drugs and for maintaining low intracellular platinum levels.

### Cells overexpressing FGF13 exhibit resistance to cisplatin/copper and lower intracellular existence of the respective metals

**Figure 3:**
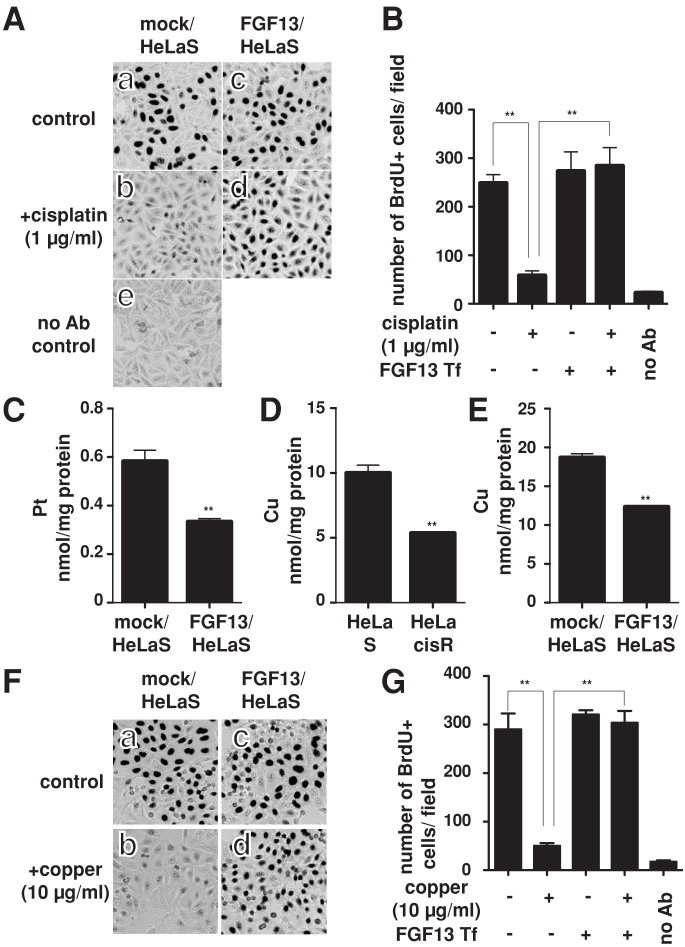
Overexpression of FGF13 provides HeLa S cells with resistance to cisplatin and copper. (A). HeLa S cells seeded on culture slides were transfected with an expression vector encoding FGF13 variant 2/3/5 (FGF13/HeLa S) or a mock vector (mock/HeLa S), and cellular resistance to 1 μg/ml cisplatin was assessed after 18 h using a Cell Proliferation kit. The BrdU-incorporated nuclei were immunodetected and visualized by DAB staining (black signals). False positive signals detected in the absence of anti-BrdU antibody were also counted and shown as the negative control [no Ab control]. (B). Numbers of BrdU-positive cells identified as in panel A were counted and plotted. Ten microscope fields were analyzed for each sample, and the averages and SD were calculated. **, p < 0.01. (C). HeLa S cells stably transfected with an FGF13 expression vector or a mock vector were cultured for 18 h in medium containing 3 μg/ml cisplatin, then harvested and sonicated. The platinum concentration in the lysates was then measured using ICP Atomic Emission Spectrometry. **, p < 0.01. (D), HeLa S and HeLa cisR cells were cultured for 18 h in medium containing 20 μg/ml copper, then harvested and sonicated. The copper concentration in the lysates was then measured using ICP Atomic Emission Spectrometry. **, p < 0.01. (E). HeLa S cells stably transfected with an FGF13 expression vector or a mock vector were treated as in (D), and the copper concentrations in the lysates were measured. **, p < 0.01. (F), (G). HeLa S cells stably transfected with an FGF13 expression vector (variant 2/3/5) or a mock vector were treated as in (A) and (B), except that copper (10 μg/ml) was added to the medium instead of cisplatin. (G). Numbers of BrdU-positive cells identified as in panel F were counted and plotted. Ten microscope fields were analyzed for each sample, and the averages and SD were calculated. **, p < 0.01.

To further examine that the overexpressed FGF13 conferred cisplatin resistance to the cells, HeLa S cells were transfected with an FGF13 expression vector (variant 2/3/5). Then using quantitative RT-PCR we confirmed that expression of FGF13 mRNA (variant 2/3/5) was much higher (>100-fold) in the transfectants than in the mock-transfected cells. Then we analyzed cellular DNA synthesis by BrdU incorporation (Fig. 3A, blackishly stained nuclei are synthesizing DNA). We found that the FGF13-overexpressing HeLa S cells acquired significant resistance to cisplatin. When cisplatin was added to the medium (1 μg/ml) for 18 h, DNA synthesis was suppressed in mock-transfected HeLa S cells (Fig. 3A, b; Fig. 3B, second column from the left), but significantly larger numbers of BrdU-positive cells were detected among FGF13-overexpressing cells (Fig. 3A, d; Fig. 3B, second column from the right). We also found that when they were exposed to cisplatin (1 μg/ml) for 18 h, the platinum concentration was kept lower in FGF13-overexpressing HeLa S cells than in the mock transfectants (Fig. 3C).

Platinum anticancer drugs are reportedly taken up by cells via a copper transporter, suppression of which could lead to cisplatin resistance^16^. It has also been reported that increased expression of copper efflux transporters mediate resistance to cisplatin in cancer cells^17^,^18^. We therefore examined copper concentration in the cells. We found that, as with platinum, intracellular copper levels were significantly lower in HeLa cisR cells exposed to 10 μg/ml copper for 18 h than in parent HeLa S cells under the same conditions (Fig. 3D). To determine whether this effect was related to upregulated FGF13 expression, we assessed the copper concentration in FGF13-overexpressing HeLa S cells. We found that after exposure to 10 μg/ml copper for 18 h, the intracellular copper concentration was lower in cells overexpressing FGF13 than in the mock transfectants (Fig. 3E). Furthermore, analysis of the DNA synthesis showed FGF13-overexpressing HeLa S cells to be more resistant to copper than mock transfectants. When the mock/HeLaS cells were exposed to 10 μg/ml copper, the number of BrdU-incorporated cells declined significantly (Fig. 3Fa, 3Fb and 3G), but the FGF13-overexpressing cells were resistant to this copper-induced cytotoxicity (Fig. 3Fd and 3G). These results clearly demonstrate that overexpression of FGF13 makes HeLa S cells resistant to copper cytotoxicity. Thus molecules regulating the intracellular copper concentration, and which may also regulate the intracellular platinum concentration, appear to be the targets of FGF13 in cisplatin resistant HeLa cisR cells.

### Association between FGF13 expression in cervical cancer biopsy samples and outcomes of chemoradiotherapy using cisplatin

**Figure 4:**
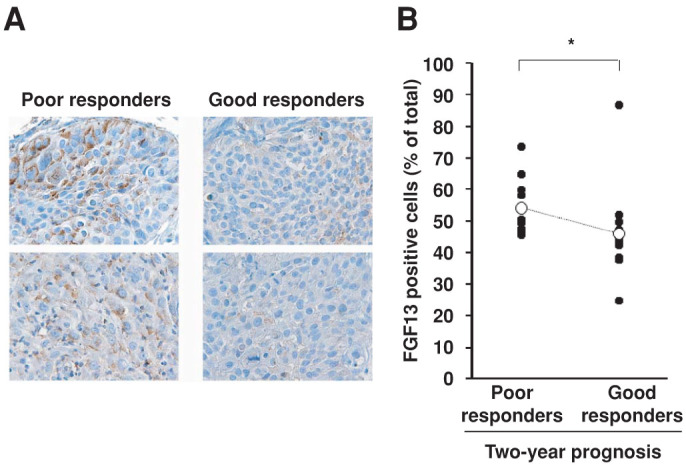
Expression of FGF13 protein in biopsy samples of cervical cancers from patients who received chemoradiotherapy using cisplatin. The subjects were 20 patients with cervical cancer. All patients received conventional chemoradiotherapy consisting of five weekly administrations of cisplatin (40 mg/m^2^). They also received radiotherapy (30.6 Gy) to the whole pelvis plus additional parametric radiation with central shielding to complete a 50.6 Gy dose, along with ^192^Ir high dose-rate intracavitary brachytherapy. Levels of FGF13 expression were analyzed using a streptavidin-biotin immunoperoxidase technique. Negative controls for all experiments (no primary antibody) were incubated in the same manner at each step. For each biopsy sample, 10 non-overlapping fields were randomly selected and analyzed, and the percentage of cells positive for FGF13 expression per total tumor cells in each field was calculated. A. Representative immunohistochemical staining of FGF13 for comparison with prognosis. Positive cell counts are expressed as the average number of cells per field. B. Percentages of FGF13-positive cells. *, p < 0.05. If two extreme results are omitted from each group, p value becomes 0.02.

We next assessed the importance of upregulated FGF13 expression for cisplatin resistance in clinical samples. Because HeLa cells were originally derived from cervical cancer, we analyzed the expression of FGF13 in biopsy samples obtained from cervical cancer patients before they received chemoradiotherapy using cisplatin (Table 3). Immunohistochemical analyses using the same anti-FGF13 antibody used in Figs. 1E, 1F and 2B showed that the percentage of FGF13-positive cells was significantly higher in tumor samples from patients with a poor two-year prognosis (i.e., poor responders to chemoradiotherapy) than in tumors from patients with a good prognosis (i.e., good responders to chemotherapy). Typical specimens from two patients in each group are shown in Fig. 4A, and the group data for all the specimens are shown in Fig. 4B. These results indicate that endogenous expression of FGF13 in cancer cells may affect the outcome of chemoradiotherapy using cisplatin. Thus the levels of FGF13 expression may serve as a useful guide when planning treatment for cervical cancer patients.

## Discussion

Our present findings indicate that upregulated FGF13 expression plays a pivotal role in the resistance of HeLa cisR cells to platinum anticancer drugs, and that the resistance reflects a reduction in the drugs' intracellular concentrations. It is widely believed that multiple molecular mechanisms underlie cisplatin resistance. In some cases cisplatin efflux may be mediated by ATP-binding cassette (ABC) transporters (ABCs) such as P-glycoprotein (i.e., ABCB1)^19^. However, we found that neither probenecid, an inhibitor of many ABCs, including P-glycoprotein, nor verapamil, a P-glycoprotein inhibitor, affected cisplatin resistance in HeLa cisR cells (Supplementary Fig. S3). This suggests these ABC transporters do not greatly contribute to cisplatin resistance in HeLa cisR cells.

Alternatively, cisplatin resistance in HeLa cisR cells could involve glutathione/SLC7A11-mediated detoxification in addition to FGF13-dependent lowering of the intracellular platinum concentration. We found that expression of SLC7A11 is strongly upregulated in both HeLa cisR and S180 cisR cells (Supplementary Fig. S4). SLC7A11 forms a complex with SLC3A2, which then acts as an amino acid transporter that exchanges intracellular glutamate for extracellular cystine. It has been suggested that the SLC7A11/SLC3A2 heterodimeric transporter mediates drug resistance by supplying cystine to cells for glutathione maintenance. Consistent with that idea, increased incorporation of cystine into cells via SLC3A2/SLC7A11 reportedly raises intracellular levels of both glutathione and glutathione S-transferase^20^,^21^. The glutathione formed then conjugates with the anticancer drugs and is exported via the glutathione conjugate export pump to achieve their detoxification^22^. We found that intracellular glutathione levels in HeLa cisR cells (35.7 μmol/mg cellular protein) were approximately three times higher than in cisplatin-sensitive HeLa S cells (12.8 μmol/mg cellular protein). This suggests that lowering intracellular cisplatin concentrations in resistant HeLa cisR cells involves not only the putative FGF13-regulated mechanism, but also upregulation of SLC7A11 and an increase in intracellular glutathione.

Our results suggest that FGF13 may act by mediating a reduction in copper/platinum uptake and/or an increase in copper/platinum efflux. Furthermore, it appears likely that FGF13 regulates not the expression level but the activity of one or more influx/efflux transporters, as mRNA expression of a copper transporter (Ctr1: SLC31A) and two copper efflux transporters (ATP7A and ATP7B) were not greatly affected in HeLa cisR cells, as compared to control cells (The fold differences in terms of the log_2_ of the expression level of SLC31A, ATP7A and ATP7B were −0.5, 1.1 and 0.2, respectively). This is not the first time it has been suggested that the function of a metal transporter/channel could be regulated by FGF13. It has also been reported that FGF13 interacts with voltage-gated sodium channels (VGSCs) and modulates their activity^23^. In addition, a structural study has shown that FGF13 binds to the C-terminal domain of VGSCs together with calmodulin^24^. We hypothesize that FGF13 may interact with one or more copper transporters and regulate their activity; however, this hypothesis awaits future study.

Several earlier studies have shown that FGF1 and FGF2, which contain structural features similar to FGF13, including the absence of a signal peptide in their primary structures, are exported or secreted from their expressing cells via a non-classical pathway^25^,^26^. It has also been reported that extracellular FGF12, a protein structurally and functionally similar to FGF13, is efficiently incorporated into cells from outside^27^. The uptake appears to be mediated by a “cell membrane permeable peptide” within the primary structure of FGF12, and once taken up FGF12 exerts a radioprotective effect. These findings predict there may be a mechanism by which FGF13 is exported from its expressing cell via a non-classical pathway and then incorporated into nearby cells, where it then exerts its biological activity.

Finally, our findings point to FGF13 as a novel target for anticancer therapy. More detailed investigation into its molecular mechanism will be important for our understanding of platinum drug resistance in cancer.

## Methods

### Establishment of cisplatin-resistant HeLa cisR cells

Parental HeLa S (a derivative of HeLa cell line; a kind gift from Dr. Handa at Tokyo Institute of Technology) cells were maintained in MEM supplemented with 10% fetal bovine serum (FBS: Cancera International, Ontario, Canada), 1% MEM-nonessential amino acids (Invitrogen, MD, USA), and 20 mM HEPES (Nissui, Tokyo, Japan). By then subculturing these cells in the presence of incrementally increasing concentrations of cisplatin, we established a highly resistant subline, HeLa cisR. Moreover, by collecting the cells at several intermediate times during the process of establishing the HeLa cisR line, we obtained intermediate lines #1, #2, #3, #4 and #5, which were adapted to 0.42, 0.84, 2.0, 6.0 and 8.0 μg/ml cisplatin, respectively. Ultimately, the #5 cells were maintained in the presence of 6.0 μg/ml cisplatin and designated HeLa cisR cells. HeLa cisR cells were confirmed to have originated from HeLa S cells by short tandem repeat (STR) analysis (performed by TAKARA Bio Inc., Osaka, Japan). The STR profile of HeLa S cells was similar to that of HeLa cells (ATCC CCL-2) and HeLa S3 cells (ATCC CCL-2.2). Cisplatin-resistant mouse sarcoma S180 cisR cells were established previously from parental S180 cells (obtained from Japanese Collection of Research Bioresources, Osaka, Japan) and maintained in MEM supplemented with 5% FBS, 1% MEM-nonessential amino acids, and 20 mM HEPES^15^. Parent S180 cells and the S180 cisR cells were verified to be mouse cells by partial nucleotide sequencing of m*Fgf13* gene.

### Measurement of cytotoxicity based on cell number

The cytotoxicity of platinum drugs was analyzed as described previously^15^. Cells (2.5 × 10^4^/ml, 180 μl/well) were seeded into 96-well plates and cultured in the presence of various concentrations of platinum drugs (added at 20 μl/well) at 37°C under 5% CO_2_. After 3 days, cytotoxicity was measured based on cell number as follows. Cell Counting Kit (CCK)-8 reagent (a modified MTT assay system; Dojin Laboratories, Tokyo, Japan, 10 μl/well) was added to each well, and the plates were incubated for 4 h, after which the absorbance at 450 nm was measured using a microplate reader (Model 680, Bio Rad Laboratories, Inc). The ratio of the cell number in the drug-containing culture versus that in the control drug-free culture was calculated.

### Measurement of cytotoxicity using BrdU incorporation as an index of DNA synthesis

As an alternative assay to measure the cytotoxicity of platinum drugs, cellular DNA synthesis was analyzed based on bromodeoxy-uridine (BrdU) incorporation using a Cell Proliferation Kit (GE Healthcare). HeLa S cells (2 × 10^4^ cells/ml, 200 μl/well) were seeded onto culture slides (BD Falcon, 8 wells/slide) and transfected the next day with a FGF13 construct (variant 2/3/5) using FuGENE HD (Roche diagnostic). On the next day cisplatin (final concentration: 1 μg/ml) was added, and the cells were incubated for 18 h, after which the medium was removed, and the slide was cultured for 1.5 h with BrdU (diluted 1:1000 by culture medium) before being fixed for 30 min with acid ethanol (acetic acid: distilled water: ethanol = 5:5:90). The fixed cells were then treated with nuclease/anti-5-bromo-2′-deoxyuridine antibody followed by peroxidase anti-mouse IgG2a and stained by 3,3′-diaminobenzidine tetrahydrochloride (DAB) according to the manufacture's protocol. The numbers of stained cells per field was counted under a microscope.

### RNA isolation and quantitative RT-PCR

Total RNA was isolated from cells using an RNeasy Mini Kit (QIAGEN, Valencia, CA). cDNA was then synthesized using 1 μg of total RNA with a First Strand cDNA Synthesis Kit (Roche Diagnostics, Indianapolis, IN, USA). The specific primer sets used for quantitative RT-PCR are shown in Table 1. The quantitative RT-PCR analyses were performed using Light Cycler Fast Start DNA SYBR Green I kit and a Light Cycler (Roche Diagnostics).

### RNA interference

We used a BLOCK-iT™ Pol II miR RNAi expression vector kit (Invitrogen) to induce RNA interference. Using FGF13BLOCK-ITmiR RNAi Select (Invitrogen), top and bottom oligo DNA was synthesized and inserted into pcDNA6.2-GW/EmGFP-miR vector (Invitrogen). The plasmid pcDNA6.2-GW/EmGFP-miR-neg, which carries a scrambled sequence and cannot target any known vertebrate gene, served as a control non-targeting vector. HeLa cisR cells were transfected with each vector using Lipofectamine 2000 (Invitrogen) and then cloned by limiting dilution to yield FGF13Kd#1, FGF13Kd#2 and mock transfectant cells.

### Polyclonal anti-FGF13 antibody

A cDNA fragment corresponding to amino acid residues 63–245 of human FGF13 variant 1 (conserved in all variants) was subcloned into pGEX-5X-1 and expressed in *E. coli* as a GST-fusion protein. Rabbits were immunized with the purified GST-FGF13, and the antiserum was affinity-purified using the same protein. The resultant polyclonal anti-FGF13 antibody was used for immunocytochemical and flow cytometric analyses.

### Flow cytometric analysis of FGF13 protein expression

The fixation and membrane permeabilization of the cells was conducted with acid ethanol for 30 min. The fixed cells were washed three times with phosphate buffered saline containing 0.1% sodium azide and 0.1% bovine serum albumin, and were incubated with affinity-purified rabbit polyclonal anti-FGF13 antibody on ice for 60 min. For negative control samples, the cells were not incubated with the primary antibody. After washing, the cells (1 × 10^6^) were incubated with 5 μg/ml fluorescein isothiocyanate (FITC)-conjugated F (ab′)_2_ goat anti-rabbit IgG (MP Biomedicals, Solon, OH) on ice for 30 min. After washing, the cells were subjected to flow cytometric analysis (Epics Elite, Beckman-Coulter Inc., Fullerton, CA).

### Measurement of intracellular platinum and copper

Intracellular platinum and copper concentrations were measured as described previously^15^. For short-time analyses (0–3 h), cells maintained in an exponential growth phase were incubated in medium containing 30 μg/ml cisplatin, then washed and sonicated (Sonifier 250, Branson). For long-time analyses (18 h), cells were incubated with 1 μg/ml cisplatin or 10 μg/ml copper. The platinum or copper concentrations in the lysates were then measured using ICP Atomic Emission Spectrometry (ULTIMA2, HORIBA, Ltd., Kyoto, Japan).

### Constructs and vectors

Human FGF13 cDNA variants 1, 2 and 3/5 were subcloned into pcDNA3.1w/(FLAG)3-(His)6^28^ and subcloned into pcDNA3.1 (Invitrogen). The sequences of the constructs were verified by DNA sequencing.

### Immunocytochemistry

HeLa S cells (2 × 10^4^/ml, 200 μl/well) were seeded onto culture slides (BD Falcon, 8 wells/slide), transfected the next day with FGF13 variant 2/3/5 using FuGENE HD (Roche diagnostic) and fixed for 30 min with acid ethanol. The fixed cells were then incubated with anti-FGF13 antibody (1/50, 150 μl/slide) for 1 h at room temperature, washed three times with PBS, and incubated with FITC-anti-rabbit IgG (1/100 dilution, 150 μl/slide, Cappel, MP Biomedicals, Solon, OH) for 1 h at room temperature. After washing the cells, the cells were counterstained using PI-mounting medium (Vectashield, Vector Laboratories, Inc. Burlingame, CA), and the slides were photographed under a fluorescence microscope (Keyence, BZ-9000).

### Measurement of intracellular GSH levels

Intracellular GSH levels were measured using a Glutathione Assay Kit (Cayman Chemical Company, Ann Arbor, MI) according to the manufacturer's instructions. HeLa S, FGF13-knockdown clones #1 and #2, and HeLa cisR cells were seeded into 6-cm dishes (2 × 10^5^ cells/dish), cultured, collected using a cell scraper and sonicated. After a small volume (100 μl) of each sample was reserved for protein assays, the samples were deproteinated using metaphosphoric acid (SIGMA). The sulfhydryl group of GSH reacts with DTNB (5,5′-dithio-bis-2(nitrobenzoic acid) to produce the yellow colored 5-thio-2-nitrobenzoic acid (TNB). The absorbance of TNB at 410 nm was measured by a plate reader (Infinite 200, TECAN Group, Ltd., Männedorf, Switzerland). Protein concentrations were measured using a BCA Protein Assay Reagent Kit (Pierce, Rockford, IL) according to the manufacturer's protocol, and the GSH concentration (μmol/mg cellular protein) was calculated.

### Statistical analysis

The significance of differences was statistically analyzed using two-way ANOVA followed by Bonferroni's multiple comparisons test (Fig. 1 to 3) or Mann-Whitney analysis (Fig. 4), using Prism software (GraphPad Prism, San Diego, CA).

### Analysis of expression of FGF13 protein in biopsy samples of cervical cancers

This study included 20 patients with cervical cancer who were treated at the National Institute of Radiological Sciences, Chiba, Japan. These patients gave appropriate informed consent to allow examination of their tissues and medical records, and the study protocols were approved by the institutional review board^29^,^30^. Clinical stage and histological classification were based on criteria of the International Federation of Gynecology and Obstetrics. All patients received conventional chemoradiotherapy consisting of five weekly administrations of cisplatin (CDDP, 40 mg/m^2^). They also received radiotherapy (30.6 Gy) to the whole pelvis plus additional parametric radiation with central shielding to complete a 50.6 Gy dose, along with ^192^Ir high dose-rate intracavitary brachytherapy. Levels of FGF13 expression were analyzed using a streptavidin-biotin immunoperoxidase technique described elsewhere^29^,^30^. Negative control specimens for all experiments (no primary antibody) were incubated in the same manner at each step. For each biopsy sample, 10 non-overlapping fields (100× objective) were randomly selected and analyzed, and the percentage of cells positive for FGF13 expression per total tumor cells in each field was calculated.

## Author Contributions

T. Okada and T. Imamura designed the entire study; T. Okada prepared HeLa cisR and S180 cisR cells; T. Okada, K.M., R.H. and C.M. performed the cellular and biochemical analyses, T. Okada, T.K., M.K. and T. Imamura supervised the in vitro experiments, M.N. performed immunohistochemical experiments, F.M., M.W. and T. Imai supervised clinical study, T. Ohno and S.K. performed isolation and interpretation of the clinical samples; T. Okada and T. Imamura wrote the manuscript.

## Supplementary Material

Supplementary Information Supplementary Information

## Figures and Tables

**Table 1:** Primer sequences used for RT-PCR

Target*	Primer sequences (5′ to 3′)	Product size (bp)
hFGF13 (all variants)	Forward 5′- ACAAGCCTGCAGCTCATTTT -3′	
	Reverse 5′- CTTTTGCCCTCACTGGCTAC -3′	192
hFGF13v1	Forward 5′- GGAGGAGCTCGGACGGCATGC -3′	
	Reverse 5′- ATAGCCGCCGCCATGGCCAC -3′	204
hFGF13v2/3/5	Forward 5′- CCCTTTCGTGCTAAGTGTCA -3′	
	Reverse 5′- CTTAAGCTGAGGCTCTTCCG -3′	92
hFGF13v4	Forward 5′- GCGGTGGGGAAAAGCGGATTC -3′	
	Reverse 5′- AAGCTGAGGCTCCTTAGAAGC -3′	197
hFGF13v6	Forward 5′- CTCTGTGTCTGCTCCAAATGT -3′	
	Reverse 5′- GGGATGAGGTTAAACAGAGTG -3′	219
hSLC7A11	Forward 5′- CTGGAGACCTCGACAGCAGTCTTTT -3′	
	Reverse 5′- ACTCCAGTCAGAGTGATGACGA -3′	238
hGAPDH	Forward 5′- CTGACCTGCCGTCTAGAAAAAC -3′	
	Reverse 5′- GTCTCTCTCTTCCTCTTGTGCTCT -3′	325
mFgf13	Forward 5′- CCTCGGAACATTTCACACCT -3′	
	Reverse 5′- AGTGGTTTGGGCAGAAAATG -3′	199
mSlc7A11	Forward 5′- TCCTCTGCCAGCTGTTATTG -3′	
	Reverse 5′- GAGAGGCACCTTGAAAGGAC -3′	184
mGapdh	Forward 5′-CCCCTTCATTGACCTCAACTAC-3′	
	Reverse 5′-TGGTGGTGAAGACACCAGTAGA-3′	209

*GAPDH, glyceraldehyde-3-phosphate dehydrogenase.

**Table 2:** Human FGF13 mRNA splice variants examined in this study

FGF13 transcript variant*	N-terminal amino acid sequence
variant 1	MAAAIASSLIRQKRQAREREKSNACKCVSSPSKGKTSCDKNKLNVFSRVKLFGSKKRRRRRP/EPQL…
variant 2	MSGKVTKPKEEKDASKVLDDAPPGTQEYIMLRQDSIQSAELKKKESPFRAKCHEIFCCPLKQVHHKENTEPE/EPQL…
variant 3/5	MLRQDSIQSAELKKKESPFRAKCHEIFCCPLKQVHHKENTEPE/EPQL…

*The NCBI page at the link below was consulted. It listed six variants of the human FGF13 transcript, encoding five isoforms of FGF13 polypeptide (transcript variants 3 and 5 encode the same isoform). Variants 4 and 6 are not expressed at detectable levels in either HeLa S or HeLa cisR cells (described in the text). Expression of variants 2/3/5 were detected using a common oligonucleotide primer that corresponds to the underlined amino acid sequence. http://www.ncbi.nlm.nih.gov/nuccore?Db=gene&DbFrom=nuccore&Cmd=Link&LinkName=nuccore_gene&IdsFromResult=21706384

**Table 3:** Patient characteristics

Clinical factors	Case No.
**Age (median, range)**	**53 (37–63)**
**FIGO stage**	
I	1
II	0
III	13
IV	6
**Histologic classification**	
Squamous carcinoma	16
Adenosquamous carcinoma	4
Outcome*	
Good responders	10
Poor responders	10

*Disease-free status (good/poor responders) 2 years after treatment. Patients who showed no evidence of disease 2 years after treatment were defined as good responders (n = 10). Patients who were alive with either a recurrent tumor or newly developed metastasis as well as patients who died because of their tumor within 2 years after treatment were defined as poor responders (n = 10). In each group, 8 patients were diagnosed with squamous cell carcinoma and 2 with adenosquamous carcinoma.
