# Supplementary material for: Upregulated expression of FGF13/FHF2 mediates resistance to platinum drugs in cervical cancer cells
Source: Sci Rep. 2013 Oct 11;3:2899. doi: 10.1038/srep02899 (PMC3795355; doi:10.1038/srep02899)

Scientific Reports

Supplementary Information

Upregulated expression of FGF13/FHF2 mediates resistance to platinum drugs in cervical cancer cells

Tomoko Okada, Kazuhiro Murata, Ryoma Hirose, Chie Matsuda, Tsunehiko Komatsu, Masahiko Ikekita, Miyako Nakawatari, Fumiaki Nakayama, Masaru Wakatsuki, Tatsuya Ohno, Shingo Kato, Takashi Imai, and Toru Imamura*

* Corresponding author: Dr. Toru Imamura, Signaling Molecules Research Group, Biomedical Research Institute, National Institute of Advanced Industrial Science and Technology (AIST)

E-mail: imamura-toru@aist.go.jp

Supplementary Table S1

Genes largely upregulated in HeLa cisR cells compared to HeLaS parent cells

accession number	gene name	expression upregulation ratio (log_2)*		gene description
		Exp. 1	Exp. 2	
NM_004114	FGF13	8.5	3.2	fibroblast growth factor 13
AF141347	TUBA1A	7.0	4.9	tubulin, alpha 1a
U13700	CASP1	6.9	4.2	caspase 1, apoptosis-related cysteine peptidase
AF264014	CD163L1	6.4	3.1	CD163 molecule-like 1
NM_001423	EMP1	6.3	2.0	epithelial membrane protein 1
NM_013233	STK39	6.3	4.7	serine threonine kinase 39 (STE20/SPS1 homolog, yeast)
AW134979	STXBP6	6.1	3.2	syntaxin binding protein 6 (amisyn)
AF494344	FAM9B	6.0	2.5	family with sequence similarity 9, member B
S73751	CES1	5.8	3.3	carboxylesterase 1 (monocyte/macrophage serine esterase 1)
BC002666	GBP1	5.8	3.3	guanylate binding protein 1, interferon-inducible, 67kDa
U13699	CASP1	5.7	4.1	caspase 1, apoptosis-related cysteine peptidase
AA083478	TRIM22	5.6	7.8	tripartite motif-containing 22
NM_052889	CARD16	5.5	4.2	caspase recruitment domain family, member 16
AI089025	SGCB	5.4	3.4	sarcoglycan, beta (43kDa dystrophin-associated glycoprotein)
N21096	STXBP6	5.3	3.4	syntaxin binding protein 6 (amisyn)
NM_002053	GBP1	5.1	4.0	guanylate binding protein 1, interferon-inducible, 67kDa
NM_014178	STXBP6	5.1	3.2	syntaxin binding protein 6 (amisyn)
AW303375	CCDC80	5.0	2.7	coiled-coil domain containing 80
NM_001759	CCND2	5.0	2.9	cyclin D2
AW173691	ACSL5	4.9	2.0	acyl-CoA synthetase long-chain family member 5
AI075407	IFIT3	4.9	3.4	interferon-induced protein with tetratricopeptide repeats 3
BE217880	IL7R	4.8	5.0	interleukin 7 receptor
AW014593	GBP1	4.7	4.3	guanylate binding protein 1, interferon-inducible, 67kDa
U29586	SGCB	4.7	3.7	sarcoglycan, beta (43kDa dystrophin-associated glycoprotein)
NM_003246	THBS1	4.6	5.1	thrombospondin 1
U13698	CASP1	4.5	3.3	caspase 1, apoptosis-related cysteine peptidase
NM_024746	HHIPL2	4.5	4.8	HHIP-like 2
BF440025	NOV	4.5	2.9	nephroblastoma overexpressed gene
NM_002535	OAS2	4.5	3.3	2'-5'-oligoadenylate synthetase 2, 69/71kDa
U62733	CPT1B	4.4	2.3	carnitine palmitoyltransferase 1B (muscle)
AA131041	IFIT2	4.4	2.9	interferon-induced protein with tetratricopeptide repeats 2
AF135794	AKT3	4.4	3.0	v-akt murine thymoma viral oncogene homolog 3
NM_006417	IFI44	4.2	3.2	interferon-induced protein 44
NM_002380	MATN2	4.1	3.1	matrilin 2
NM_005101	ISG15	4.0	3.7	ISG15 ubiquitin-like modifier
AI678717	SGCB	4.0	3.1	sarcoglycan, beta (43kDa dystrophin-associated glycoprotein)
AA488687	SLC7A11	4.0	4.4	solute carrier family 7, member 11
NM_014331	SLC7A11	4.0	4.5	solute carrier family 7, member 11

* Genes that showed upregulated expression in HeLa cisR cells as compared to HeLa S cells [more than 16 fold (4 as the log_2 ratio) in Experiment 1] are listed in this table. Results from two separate experiments (Exp. 1 and Exp. 2, which were independent in both the cell culture and microarray analysis) are listed.

Supplementary Fig. S1.

Detection of FGF13 protein using a specific anti-FGF13 antibody in HEK transfectants

HEK293FT (Invitrogen; HEK) cells were transfected with an expression vector encoding FGF13. After two days, the cells were lysed, and the total cellular proteins were resolved by SDS-PAGE and blotted onto a PVDF membrane. After blocking, the membrane was probed with the anti-FGF13 antibody that was used throughout this study. The signal was visualized using ECL Prime Western Blotting Detection Reagent (GE Healthcare) and recorded using ChemiDoc XRS (Bio Rad Laboratories). As controls, intact HEK cells and HEK cells transfected with empty vector were similarly lysed and subjected to SDS-PAGE and Western blotting. Lane 1, HEK cells; lane 2, FGF13-transfected HEK cells; lane 3, mock-transfected HEK cells. The positions of molecular weight markers and FGF13 are indicated on the left and right, respectively.

Supplementary Fig. S1

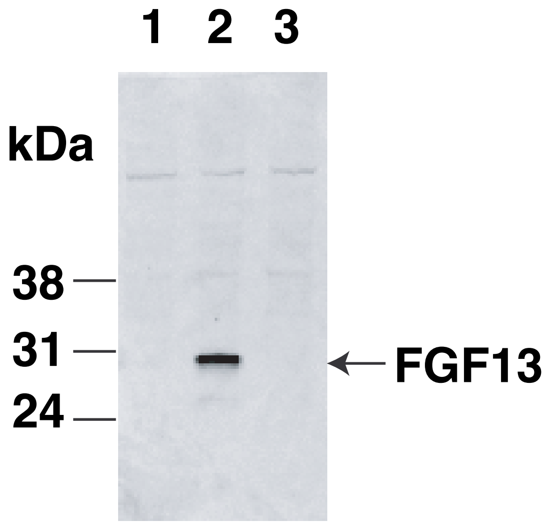

1: HEK cells
2: FGF13/ HEK cells
3: mock/ HEK cells

Supplementary Fig. S2.

Knocking down FGF13 expression restores platinum drug susceptibility to HeLa cisR cells

A, B, C. Suppressing FGF13 expression made HeLa cisR cells susceptible to cisplatin (A), carboplatin (B) and oxaliplatin (C). HeLa cisR, open squares solid line; HeLa S, filled circles solid line; FGF13Kd#2, open reverse triangles dotted line. ***, $p < 0.001$ Experiments were performed as in the legend to Fig. 2. Note that panels A and B represent the same experiment shown as Fig. 2C and D (which do not show results of Kd#2), respectively. The results with HeLa cisR and HeLa S cells are thus identical to those shown in Fig. 2. Panel C depicts an experiment independent from that shown in Fig. 2E.

Supplementary Fig. S2

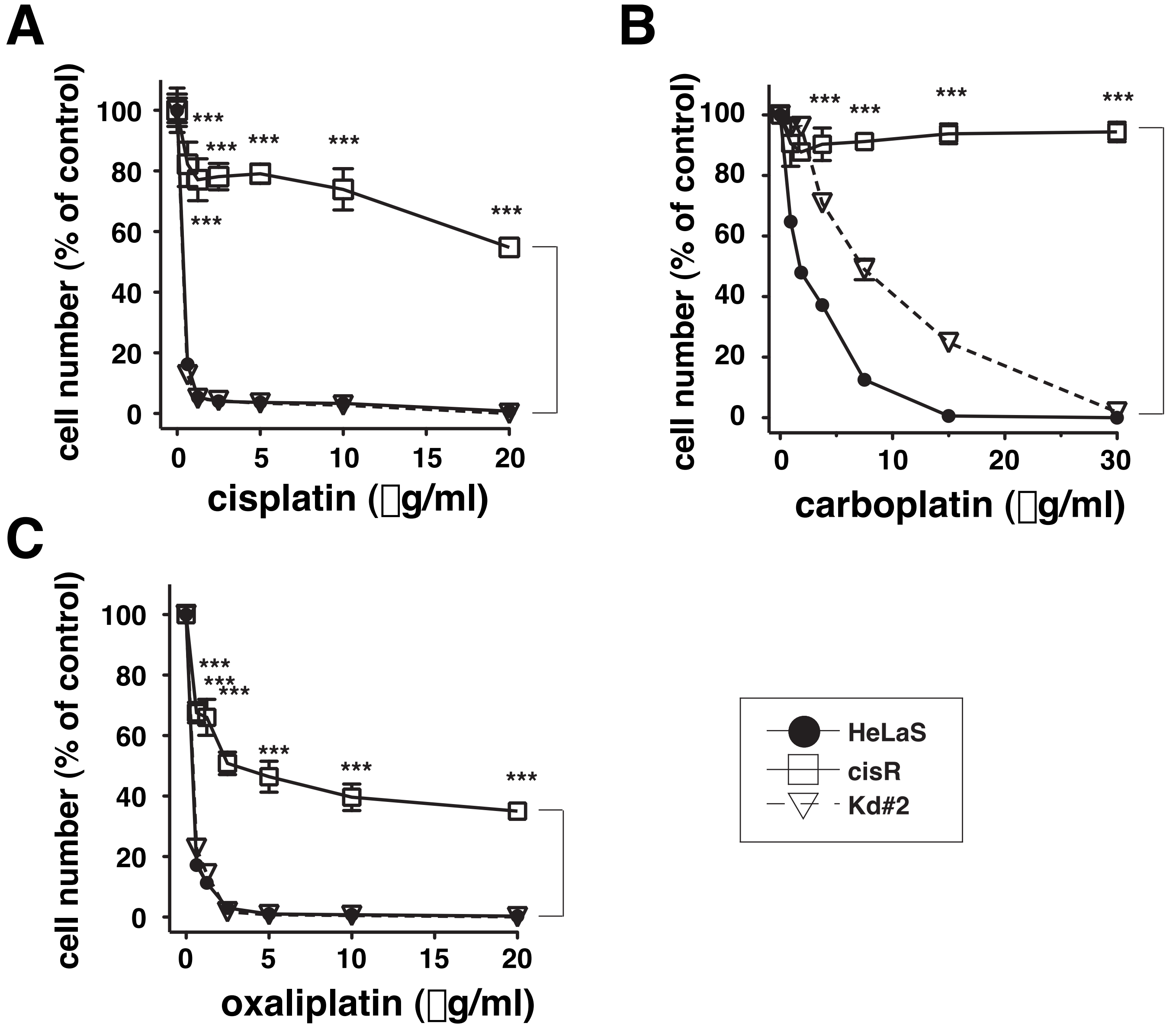

Supplementary Fig. S3

Neither probenecid nor verapamil affects cisplatin resistance in HeLa cisR cells

Effects of probenecid, an inhibitor of many ABCs including P-glycoprotein, and verapamil, a P-glycoprotein inhibitor, on the cisplatin resistance of HeLa cisR cells, or on the cisplatin sensitivity of HeLa S cells, were examined. HeLa cisR or S cells were preincubated for 1 h with the indicated concentrations of probenecid (**A**) or verapamil (**B**) before culture in the presence of the indicated concentrations of cisplatin. These concentrations of inhibitor have been shown to effectively suppress P-glycoprotein-dependent cisplatin resistance of S180 cisR cells (reference 15). After 3 days, cytotoxicity was measured based on cell number, as described in the legend to Fig. 1B. Triplicate samples were analyzed for each condition, and mean +/- S.D. are presented.

Supplementary Fig. S3

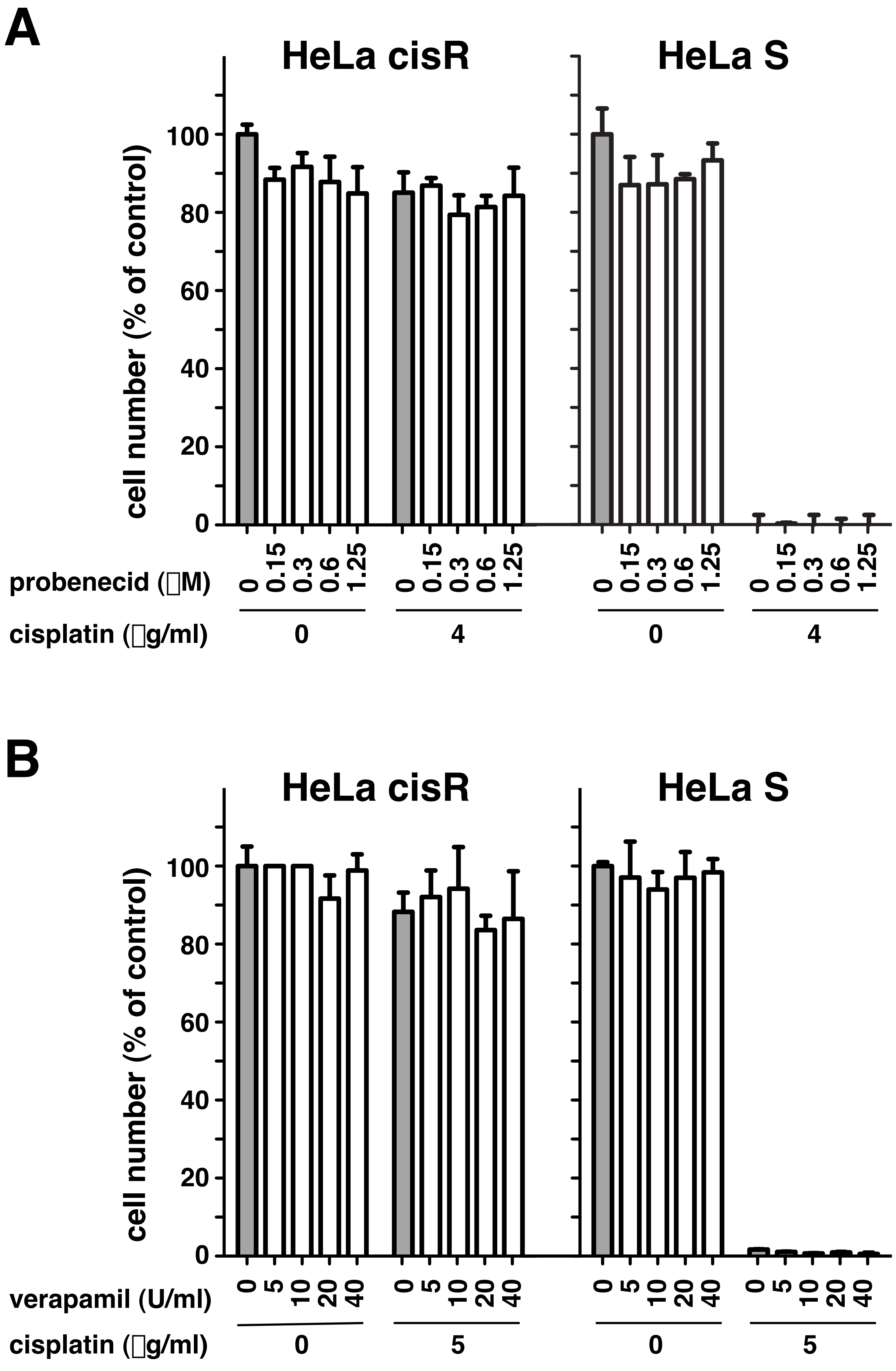

Supplementary Fig. S4

Expression of SLC7A11 mRNA is upregulated in both HeLa cisR and S180 cisR cells

A, Upregulated expression of human (h) SLC7A11 mRNA (**A**) in HeLa cisR cells as compared to HeLa S cells. **B,** Upregulated expression of mouse (m) SLC7A11 mRNA in S180 cisR cells as compared to S180 cells. The levels of SLC7A11 mRNA were quantified using the primers listed in Table 1. **, $p < 0.01$.

Supplementary Fig. S4

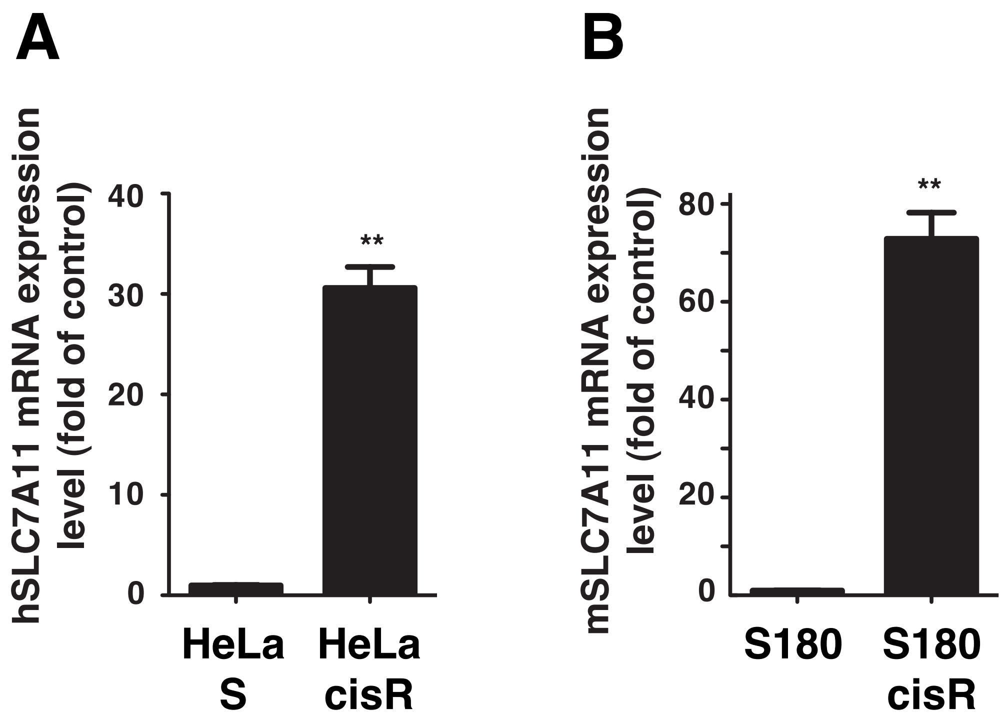

Supplement: Supplementary Information [file srep02899-s1.pdf]
